# Pathogenic Roles of Polyketide Synthase CLPKS18 and (*R*)-(-)-Mellein from *Curvularia lunata* in Maize Leaf Spot

**DOI:** 10.3390/jof11090627

**Published:** 2025-08-26

**Authors:** Zhixiang Lu, Lin Shang, Shaoqing Wang, Xinhua Wang, Yaqian Li, Shunping Zhang, Jing Wang, Jie Chen

**Affiliations:** 1 School of Agriculture and Biology, Shanghai Jiao Tong University, Shanghai 200240, China; luzhixiang@sjtu.edu.cn (Z.L.); 18846082145@163.com (L.S.); 1214900346@163.com (S.W.); xhwang@sjtu.edu.cn (X.W.); yaqianli2008@163.com (Y.L.); 2 National Key Laboratory of Non-Food Biomass Energy Technology, National Engineering Research Center for Non-Food Biorefinery, Guangxi Academy of Sciences, Nanning 530007, China; 3 The State Key Laboratory of Microbial Metabolism, Shanghai Jiao Tong University, Shanghai 200240, China; 4 Inner Mongolia Research Institute, Shanghai Jiao Tong University, Hohhot 010010, China; zsp317@163.com; 5 College of Agriculture, Hetao University, Bayannur 015000, China

**Keywords:** *Curvularia* leaf spot in maize, virulence, polyketone synthase CLPKS18, (*R*)-(-)-mellein, defense signaling pathway

## Abstract

*Curvularia lunata* (Wakkre) Boedijn is an important pathogenic fungus that causes maize leaf spot, a prevalent disease that caused significant yield losses in maize-growing areas in China in the 1990s. *Clpks18*, a polyketide synthase (CLPKS18) gene, has been identified as a crucial virulence-related gene in *C. lunata*. However, the impact of *Clpks18* and its biosynthesized virulence factor (*R*)-(-)-mellein on the expression of maize genes related to the defense signal pathway has never been determined. In this study, it was found that *Clpks18* and (*R*)-(-)-mellein significantly interfere with the signaling pathways of JA and IAA in maize leaves but in different ways and in a time-dependent manner. While CLPKS18 inhibited the maize’s JA and IAA signaling pathways through its related secondary metabolite, (*R*)-(-)-mellein inhibited the JA signaling pathway but stimulated IAA accumulation in maize leaves. In summary, understanding this novel virulence effector’s mechanism of interference with maize resistance enriches the pathology of *Curvularia* leaf spot in maize on the one hand and provides a foundation for screening the resistance germplasm and chemical fungicides against the disease on the other.

## 1. Introduction

**Figure A1:**
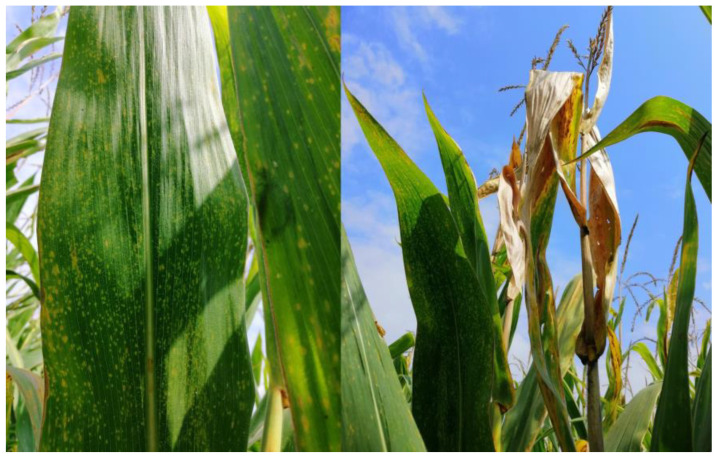
Symptoms of *Curvularia lunata* on maize leaves.

**Figure A2:**
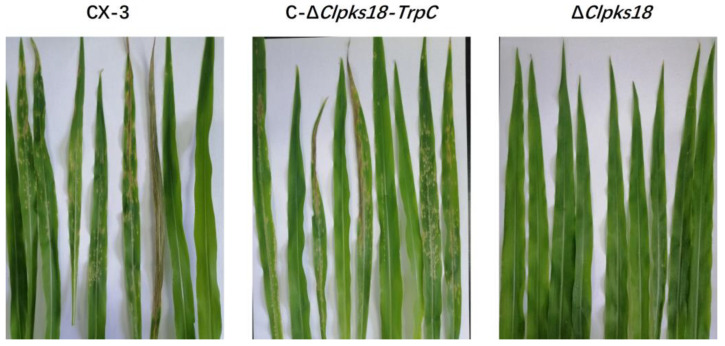
Symptoms on maize leaves treated with CX-3, ∆*Clpks18*, and C-Δ*Clpks18-TrpC* strains. Maize leaves inoculated with the wild-type strain (CX-3) and the *Clpks18*-complemented strain (C-Δ*Clpks18-TrpC*) showed significantly larger lesion areas than leaves inoculated with the *Clpks18* deletion mutant strain.

*Curvularia* leaf spot (CLS) in maize, also known as yellow spot disease, is one of the most important maize diseases worldwide. Its occurrence has been reported in Europe, Africa, Asia, and America and led to significant maize yield loss in China in the 1990s. CLS, which poses a significant threat to the yield and quality of important crops such as corn and wheat, is mainly caused by *Curvularia lunata* (Wakker) Boedijn (*C. lunata*). Field studies characterize CLS by distinct “eyespot” lesions on leaves: initial water-soaked chlorotic spots evolve into elliptical gray-brown necrotic areas (2–5 mm × 1–7 mm) with white/tan centers, dark brown margins, and yellow halos. Under high humidity, lesions produce gray-black conidia, and severe infections cause premature leaf death, reducing the yield of maize [1,2] (Figure A1). The pathogenicity mechanism of phytopathogenic fungi in host plants involves multiple stages of the pathogenic process, each of which has a corresponding major virulence factor. Currently, several pathogenicity factors have been shown to be closely related to *C. lunata* infestation in maize, such as cell wall-degrading enzymes, toxins, melanin, etc. [3,4,5]. Our secondary metabolome analysis comparing the pathogenicity-deficient strain Δ*Clpks18* with wild-type CX-3 revealed significantly impaired synthesis of the conserved virulence metabolite (*R*)-(-)-mellein in Δ*Clpks18* (Figure A2). This phytotoxin demonstrates broad functional conservation across phylogenetically diverse phytopathogens: its pathogenic role was first documented in 1986 when isolated from *Nectria fuckeliana* [6], with subsequent identification in *Stagonospora apocynin* (causal agent of cannabis leaf spot) as a necrosis-inducing agent in nine weed species including *Setaria faberii* [7]. Further studies established (*R*)-(-)-mellein as a secreted virulence determinant during *Botryosphaeria dothidea* fruit/branch infestation [8], while quantitative analyses showed its accumulation positively correlates with *Diplodia seriata* density in grapevines [9]. Critically, preliminary evidence confirms its involvement in *C. lunata* pathogenicity [10], though maize-specific mechanisms remain uncharacterized. Biosynthetically, (*R*)-(-)-mellein production requires evolutionarily conserved polyketide synthase (PKS) activity, exemplified by SNOG_00477-encoded PKS in *Parastagonospora nodorum* [11], with PKS homologs governing virulence in *Cochliobolus* spp. and *Setosphaeria turcica* [12].

**Figure A3:**
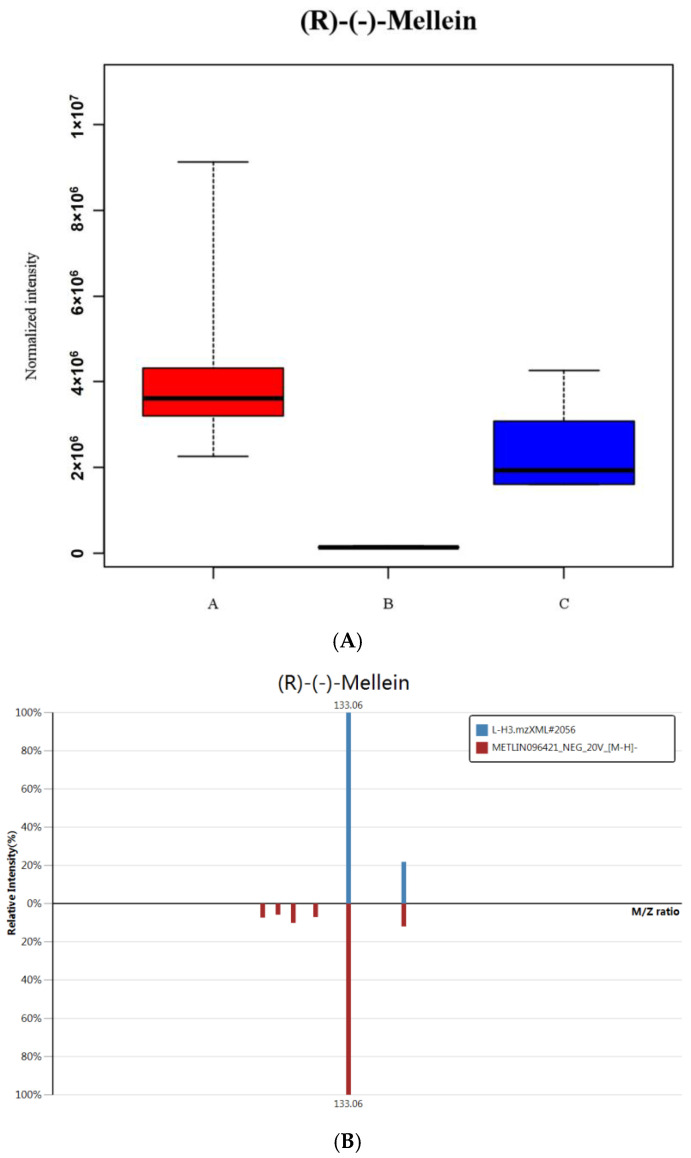
Relative quantification of (*R*)-(-)-mellein in metabolomic analysis. (**A**) The relative content of (*R*)-(-)-mellein in metabolomic analysis. Note: Analysis of the secretory metabolome showed that both the wild-type strain (CX-3, A) and the Clpks18-complemented strain (C-ΔClpks18-TrpC, C) produced (*R*)-(-)-mellein, whereas the Clpks18 deletion mutant strain (ΔClpks18, B) barely produced (*R*)-(-)-mellein. (**B**) Mass spectrometry comparison in negative ion mode. Note: The blue mass spectrum above is the spectrum of (*R*)-(-)-mellein in the standard substance library, and the red mass spectrum below is the secondary fragment spectrum of the substance to be identified in the test sample.

Our previous study showed that the *Clpks18* of *C. lunata* is strongly associated with the latter’s pathogenicity; furthermore, it has been found to be correlated with (*R*)-(-)-mellein production (Figure A2 and Figure A3). So far, there have been no reports on how (*R*)-(-)-mellein functions as a virulence factor in maize pathogenic fungi. It is also still unclear how CLPKS18 and (*R*)-(-)-mellein affect maize growth and defense responses. Phytohormones are important in various plant growth and development processes and environmental responses. Specifically, in the defense response against plant pathogen infection, salicylic acid (SA) and jasmonic acid (JA) are commonly viewed as crucial defense signals [13] that regulate host resistance against pathogen infection [14]. Therefore, in this study, we attempted to clarify the effects of the CLPKS18 and (*R*)-(-)-mellein produced by *C. lunata* on the plant hormone signaling pathways of JA, SA and IAA in maize leaves at the transcriptome level, and we also investigated the effect of (*R*)-(-)-mellein as a virulence factor on immune response- and growth-related gene expression in maize, with the aim of laying a theoretical foundation for revealing the disease-causing mechanism of (*R*)-(-)-mellein at the molecular level.

## 2. Materials and Methods

### 2.1. Strains and Maize Cultivars

*C. lunata* CX-3 was used as the wild-type strain in this study. CX-3 is a strong pathogenic strain of *C. lunata*. The pathogenicity-deficient mutant ∆*Clpks18* (∆*Clpks18* is a knockout mutant of *pks18* and C-Δ*Clpks18-TrpC*, where the latter is a complementary mutant of Δ*Clpks18*) was previously characterized by us. C-Δ*Clpks18-TrpC* is a complementary transformant of *pks18* with the *TrpC* promoter [10]. All strains were cultured in PDA medium.

Maize seeds of the varieties ZHENGDAN 958 (resistance) and Woyu 3 (susceptible) were provided by the Institute of Plant Protection, Hebei Academy of Agricultural and Forestry Sciences. Maize varieties ZHENDAN 958 and Woyu 3 are kinds of hybrids instead of pure lines or inbred lines. However, in the field growing practice, based on data from “Technical Code for Evaluation of Maize Resistance to Diseases (NY/T 1248.10-2016 [15], China)”, ZHENDAN 958 is shown to be a moderately susceptible variety, and Woyu 3 a moderately resistant variety.

### 2.2. Transcriptome Sequencing and Analysis

Each mutant and WT strain of *C. lunata* was cultured in PDA for 7 days. Conidia were suspended at a concentration of 1.0 × 10^6^ spores/ mL in a 0.05% Tween-20 solution, and 100 μL of each strain was sprayed on the leaves of the ZHENGDAN 958 maize seedlings in the five-leaf stage. The inoculated plants were kept in a growth chamber at 27 °C and in the light for 12  h. Three days post-inoculation, three leaf samples were collected from each treatment group, stored in liquid nitrogen, frozen and sent to Shanghai Parsonage Biotechnology Co., Ltd. (Shanghai, China), for eukaryotic reference transcriptome analysis (NCBI: GCF_902167145.1).

The original sequencing data were filtered, eliminating adapter sequences and low-quality ends of reads, to obtain clean data. The clean reads were aligned with the reference genome by using Hisat 2 v2.1.0 software. For RNAseq analysis, the expression levels of genes were calculated by counting the number of clean reads that could be compared to the reference genome. FPKM was used to standardize the expression levels of the reference transcriptome to make the gene expression levels of different genes and samples comparable. It is generally accepted that genes with FPKM > 1 are expressed. Then, DESeq was used to analyze the significance of differential gene expression in CX-3 and ∆*Clpks18*. The threshold conditions for DEGs were set to p-adjust (q-value) ≤ 0.05 and |Log2FoldChange| ≥ 1. The GO and KEGG databases were used for the enrichment and annotation analyses of the DEGs. The enrichment analysis method was hypergeometric distribution, with a q-value ≤ 0.05 taken as the threshold screening condition for significant enrichment of GO terms and KEGG pathways.

### 2.3. Real-Time Quantitative Polymerase Chain Reaction (RT-qPCR) Verification

The leaves of the ZHENGDAN 958 and Woyu 3 maize seedlings in the five-leaf stage were inoculated through the smearing of the conidial suspensions (1.0 × 10^6^ conidia/mL) with 0.05% Tween-20 (CX-3, C-Δ*Clpks18-TrpC* and Δ*Clpks18*). On the 3rd day post-inoculation, five inoculated leaves were randomly collected from each treatment group. Then, total RNA was extracted from the inoculated leaves by using the RNAprep Pure Polysaccharide Polyphenol Plant Total RNA Extraction Kit (Tiangen Biochemical Technology (Beijing, China) Co., Ltd.) and reverse-transcribed into DNA by using the PrimeScript™ RT reagent Kit with gDNA Eraser (Takara Biomedical Technology (Beijing, China) Co., Ltd.). RT-qPCR was carried out by using a Hieff^®^ qPCR SYBR Green Master Mix (No Rox) kit (code:11201ES08; YEASEN, Shanghai, China). The PCR was performed according to the instructions of the kit above by using a Light Cycler 96 Real-Time PCR System (Roch, Monroe County, NY, USA). *ZmUBI* was used as an internal reference to normalize the gene expression levels. The formula of 2^−ΔΔCT^ was used to calculate the relative gene expression levels. The RT-qPCR primers used in this study are shown in Table A1. Each experiment was carried out in triplicate. The multiple comparisons between groups in Section 2.2 and Section 2.3 were all conducted using Tukey’s multiple comparison test.

### 2.4. Extraction and Detection of JA, SA and IAA in Maize Leaves

The inoculated maize leaves were collected and then ground into powder by using a tissue homogenizer. A volume of 250 μL of extraction solution (methanol–acetonitrile–water–acetic acid = 40%:40%:19%:1%, *v*/*v*/*v*/*v*) was mixed with tissue powders, the mixture was centrifuged for 10 min, and the supernatant was transferred into a new EP tube; then, another 250 μL of extraction solution was added to the pellet and centrifuged again, and the supernatants from the two centrifugations were mixed and centrifuged once more for the final supernatant to be collected. JA, SA and IAA were analyzed with UHPLC-MS (30A/Sciex Quadrupole 5500, Waters Corporation (Milford, MA, USA)) in linear gradient elution mode (0~0.5 min, 10% CAN; 0.5~3 min, 10%~50% CAN; 3~4 min, 50%~100% CAN; 4~6 min, 100% CAN; 6~6.1 min, 100% ~10% CAN; 6.1~8 min, 10% CAN).

### 2.5. Signaling Pathway-Related Gene Analysis

In the previous research study, it was found that the amount of (*R*)-(-)-mellein detected in CX-3-infested maize leaves was 85.5 ng per gram [10]. Therefore, for the pathogenicity complementation assay, we inoculated maize leaves with Δ*Clpks18* conidial suspension mixed with 800 ng/mL (*R*)-(-)-mellein (final concentration 0.8 ng/μL). At the five-leaf stage of ZHENGDAN 958 maize seedlings, prepared suspensions were spot-inoculated onto leaves: CX-3, Δ*Clpks18*, C-Δ*Clpks18-TrpC* and ∆*Clpks18* + 0.8 ng/μL (*R*)-(-)-mellein. Furthermore, 0.8 ng/μL (*R*)-(-)-mellein only was inoculated in leaves as a positive control and water as a blank control. A volume of 100 uL was used for each plant inoculation. The inoculated leaves were collected at 0, 12, and 48 h post-inoculation (hpi) for the detection of the expression of genes related to the signaling pathway. The primers are listed in Table A1.

## 3. Results

### 3.1. Different Expression Patterns Between the CX-3 and ΔClpks18 Treatments

**Figure 1:**
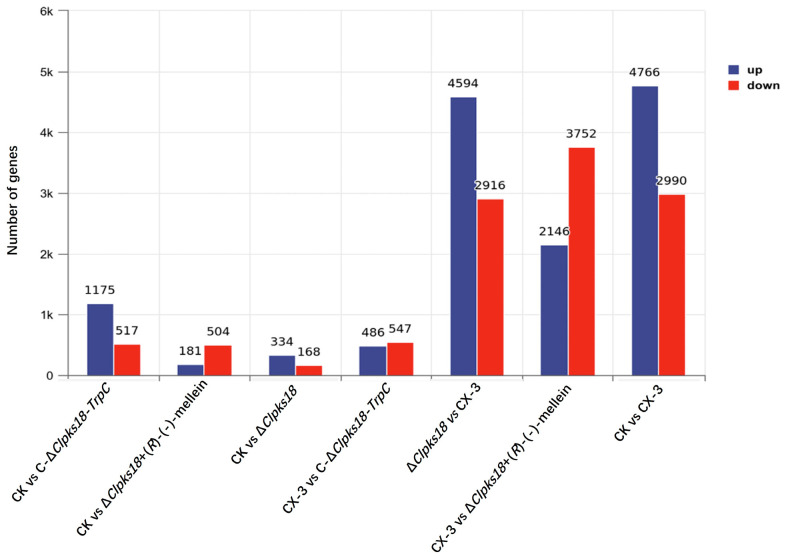
Statistics of differentially expressed genes.

Based on the cluster analysis of the expression patterns of all the samples, it was found that the three biological replicates of maize leaves inoculated with the CX-3 strain and one of the treatments of maize leaves inoculated with the C-Δ*Clpks18-TrpC* strain were clustered in a single branch and that the expression patterns of the other two biological replicates of the C-Δ*Clpks18-TrpC* strain were clustered in a branch different from the other treatments mentioned above. At the same time, we observed a relatively large difference in the severity of the disease among the three biological replicates of maize leaves inoculated with the C-Δ*Clpks18-TrpC* strain, whereas the disease was more homogeneous across the three biological replicates of maize leaves inoculated with the CX-3 strain. Therefore, we chose the more representative CX-3 treatment group for the next step of difference analysis with the Δ*Clpks18* treatment group in the transcriptome analyses. Differentially expressed genes between maize seedling leaves uninoculated and inoculated with the CX-3 strain (CK vs. CX-3) or Δ*Clpks18* (CK vs. Δ*Clpks18*) were identified by using the thresholds of *p* < 0.05 and |log2FoldChange| > 1. In response to the fungal infection, a total of 7756 genes showed differential expression at three time points post-inoculation, of which 4766 were up-regulated and 2990 were down-regulated in CK vs. CX-3. A total of 512 differential genes were observed in CK vs. Δ*Clpks18*, and 7510 (4594 up-regulated and 2916 down-regulated) differential genes were observed in Δ*Clpks18* vs. CX-3 (Figure 1). Combined with the results of the pre-pathogenicity assay [10], it can be deduced that due to the weak pathogenicity of the Δ*Clpks18* strain, it did not significantly affect the response of the host genes when it infested the host leaves. Compared with the Δ*Clpks18* strain, the CX-3 strain was able to significantly affect the response of host genes.

**Figure 2:**
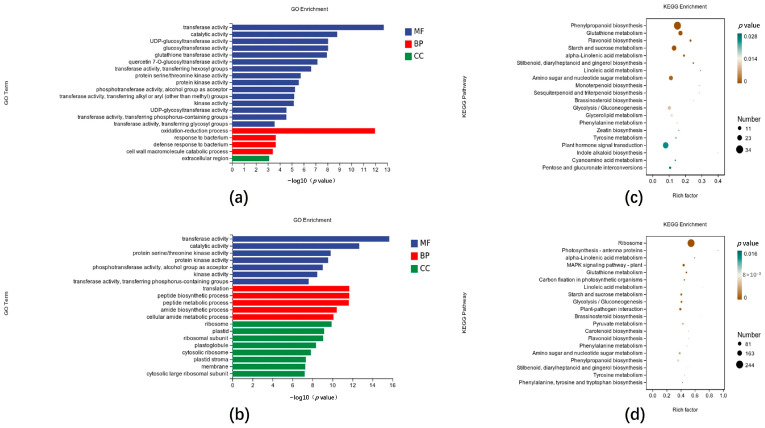
GO and KEGG analyses of differentially expressed genes. GO annotated classification of (**a**) CK vs. C-Δ*Clpks18-TrpC* and (**b**) CK vs. CX-3; enrichment factors in (**c**) CK vs. C-Δ*Clpks18-TrpC* and (**d**) CK vs. CX-3 according to KEGG annotated classification. Notes: BP (biological processes); MF (molecular functions); CC (cellular component); CK: corn not inoculated with pathogen, CX-3: corn inoculated with CX-3 strains (wild-type), C-Δ*Clpks18-TrpC*: corn inoculated with C-Δ*Clpks18-TrpC* strains.

The enrichment analysis and functional annotation of differentially expressed genes based on the GO and KEGG databases revealed that the number of genes with different expression changes in different biological processes and metabolic pathways differed. The Gene Ontology classification analysis results of the DEGs in CK vs. CX-3 and CK vs. C-Δ*Clpks18-TrpC* are shown in Figure 2. The results show that among the biological processes, response and defense response to pathogens were significantly enriched in CK vs. C-Δ*Clpks18-TrpC*. The most significantly enriched molecular functions were transfer activity and catalytic activity, and the most abundant DEGs were enriched in extracellular secretion (Figure 2a). In CK vs. CX-3, the biological processes mainly enriched were protein translation, peptide biosynthetic process, and peptide metabolic process; the molecular functions mainly enriched were transfer activity and catalytic activity; the DEGs were enriched in cytoplasm and ribosomes (Figure 2b).

**Figure A4:**
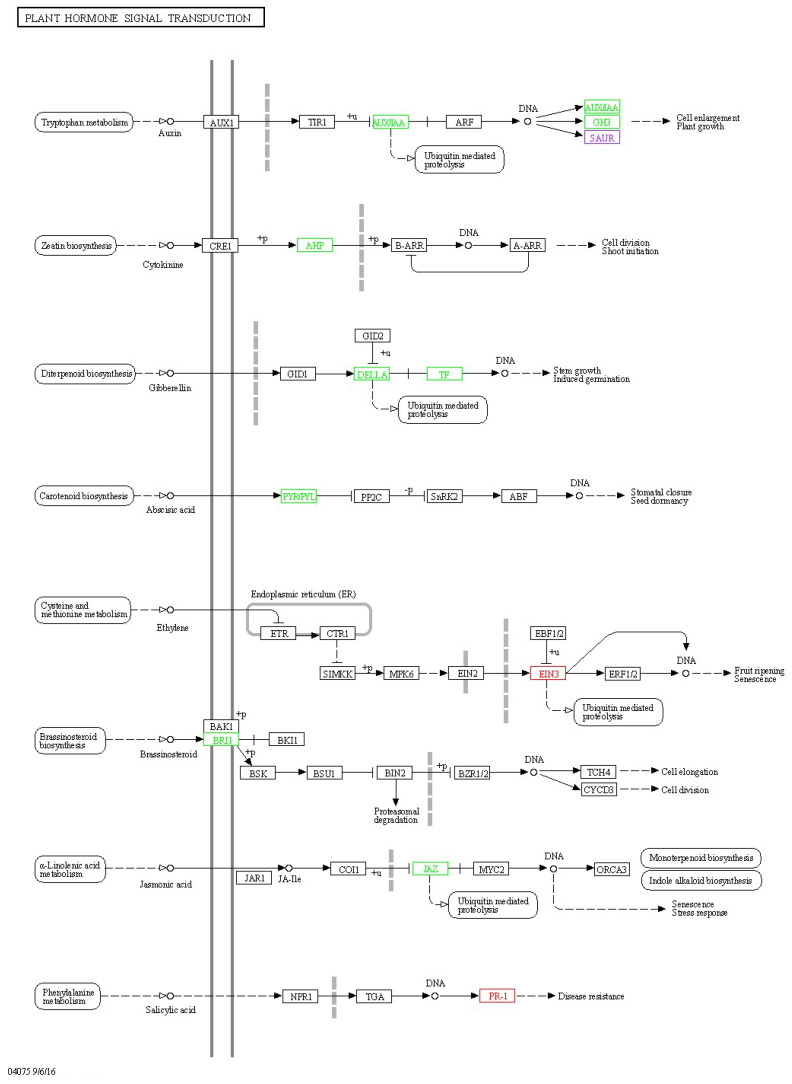
Plant hormone signal transduction in CK vs. C-Δ*Clpks18-TrpC* according to KEGG classification. Note: Green indicates that the gene is down-regulated, red indicates that the gene is up-regulated, and purple indicates that the transcript of the gene is both up- and down-regulated in the sample.

KEGG enrichment analyses were conducted in CK (corn not inoculated with pathogen) vs. C-Δ*Clpks18-TrpC* (corn inoculated with C-Δ*Clpks18-TrpC* strains), CK vs. CX-3 (corn inoculated with CX-3 strains (wild-type)) and CK vs. Δ*Clpks18* to elucidate the major metabolic pathways involved in the DEGs responding to different mutant strains of *C. lunata* in maize. In CK vs. C-Δ*Clpks18-TrpC*, the DEGs were significantly enriched in the plant hormone signaling pathway (Figure 2c). This indicates that the plant hormone signaling system of the corn inoculated with strain C-Δ*Clpks18-TrpC* has undergone significant changes compared with that of the uninoculated corn. In CK vs. CX-3, the DEGs were the most significantly enriched in the MAPK signaling pathway: plant and plant–pathogen interactions (Figure 2d). This shows that the expression of MAPK signaling pathway and genes related to the interaction between plants and pathogen in the corn inoculated with the CX-3 strain has undergone significant changes, compared with the uninoculated corn. However, the differences in the changes in the above pathways were not significant in CK vs. Δ*Clpks18*. Therefore, a subset of genes with the same expression patterns in the CK vs. CX-3 and CK vs. C-Δ*Clpks18-TrpC* transcriptomes was selected for subsequent RT-qPCR validation. The KEGG analysis of the CK vs. CX-3, CK vs. C-Δ*Clpks18-TrpC* and CK vs. Δ*Clpks18* comparative transcriptomes showed that *Clpks18* expression in *C. lunata* significantly inhibited several phytohormone signaling pathways in maize, including IAA and GA, when it infected maize leaves (Figure A4).

### 3.2. Quantitative PCR Validation Is Consistent with Transcriptome

**Figure 3:**
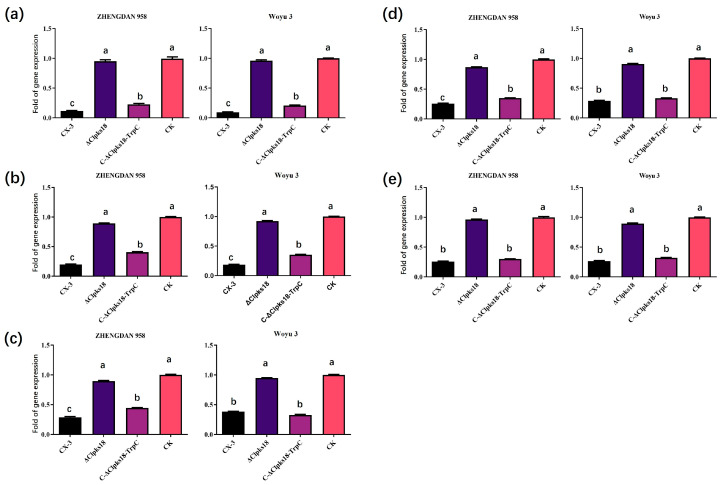
Effects of Clpks18 on maize growth hormone and gibberellin signaling pathways. (**a**) Expression of genes encoding Aux/IAA in each treatment group. (**b**) Expression of genes encoding SAUR in each treatment group. (**c**) Expression of gene encoding GH3 in each treatment group. (**d**) Expression of gene encoding PIF3 in each treatment group. (**e**) Expression of gene encoding PIF4 in each treatment group. Different lowercase letters indicate significant differences at *p* < 0.05. Bars represent the standard errors. Multiple comparisons of groups of samples by the post hoc ANOVA Fisher’s test.

The transcriptome analysis showed that the growth hormone signaling pathway was significantly repressed in ZHENGDAN 958 maize leaves inoculated with both CX-3 and C-Δ*Clpks18-TrpC*. The expression of three genes responsible for encoding plant growth hormone response proteins was analyzed by using RT-qPCR validation to confirm the reliability of the generated RNA-seq data. The selected genes were AUX/IAA (K14484, LOC100274569), SAUR (Small auxin-up RNA, K14488, LOC103653849) and GH3 (auxin-responsive GH3 gene family, K14487, LOC100193303). The RT-qPCR results showed that the expression of the three genes, *AUX/IAA*, *SAUR* and *GH3*, was significantly down-regulated in maize leaves inoculated with the CX-3 and C-Δ*Clpks18-TrpC* strains 72 h post-inoculation, in both the susceptible cultivar, Woyu 3, and the disease-resistant cultivar, ZHENGDAN 958. In contrast, the expression of the above three genes in Δ*Clpks18*-inoculated leaves was not significantly different from the control (Figure 3a–c). The RT-qPCR results show that the expression trends of the genes were consistent with the RNA-Seq data. The results of the RT-qPCR of two photopigment interactors, *PIF3* and *PIF4*, are consistent with the transcriptome sequencing results: the expression of *Clpks18* in *C. lunata* was found to significantly repress the expression of the phytochrome-interacting factors, *PIF3* and *PIF4* in both susceptible and resistant maize leaves (Figure 3d,e). In conclusion, it is shown that the pathogen *Clpks18* significantly inhibits the expression of the AUX/IAA and gibberellin signaling pathway-related genes in maize during *C. lunata* infestation.

### 3.3. Effects of Clpks18 on Hormone Production and Signaling Systems in Maize

**Figure 4:**
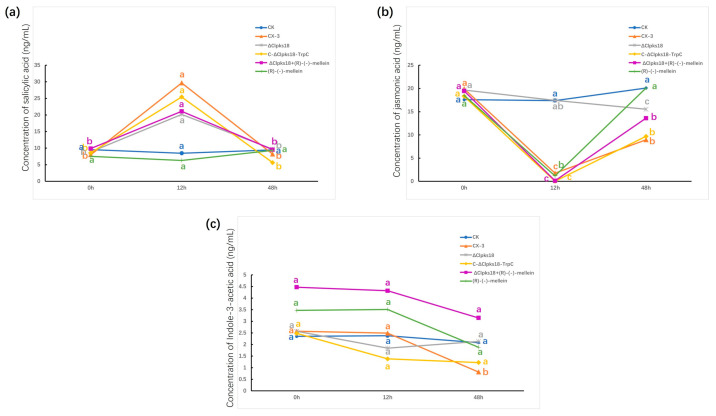
Contents of SA, JA and IAA in leaves for each inoculation treatment at different times. (**a**) Content of SA in leaves for each inoculation treatment; (**b**) content of JA in leaves for each inoculation treatment; (**c**) content of IAA in leaves for each inoculation treatment. Each treatment was repeated three times. Letters represent conditions with significant differences according to the post hoc ANOVA Fisher’s test (*p* < 0.05).

We next inoculated ZHENGDAN 958 maize leaves with spore suspensions of the strains CX-3, Δ*Clpks18* and C-Δ*Clpks18-TrpC* and sampled them on the 0th, 12th and 48th hour post-inoculation to explore how *Clpks18* affects the production of maize hormones. Then, we determined the concentrations of SA, JA and IAA in the leaves of plants subjected to different treatments with UHPLC-MS. At 0 h post-inoculation, there were no significant differences in the contents of SA, JA and IAA among the treatments (Figure 4). This indicates that the initial SA, JA and IAA levels before inoculation were the same, and the physiological status was similar among plants in each treatment. At 12 h post-inoculation (12–24 h), the content of SA was significantly increased in maize leaves inoculated with CX-3, Δ*Clpks18* and C-Δ*Clpks18-TrpC* compared with CK (Figure 4a), and the content of JA was significantly decreased in maize leaves inoculated with CX-3 and C-Δ*Clpks18-TrpC* (Figure 4b). In addition, the content of IAA in the three treatments was not significantly different from that of CK (Figure 4c). At 48 h post-inoculation, the content of SA in the leaves of the three treatments recovered to a level that was not significantly different from that of CK (Figure 4a); the content of JA in CX-3- and C-Δ*Clpks18-TrpC*-inoculated maize leaves was increased to a level that was about half of that of CK (Figure 4b); and the content of IAA decreased slightly, with its average dropping to about half of that of CK (Figure 4c). However, the difference did not reach the 95% significant level compared with CK. The results reveal that *Clpks18* mainly interferes with host JA synthesis.

**Figure 5:**
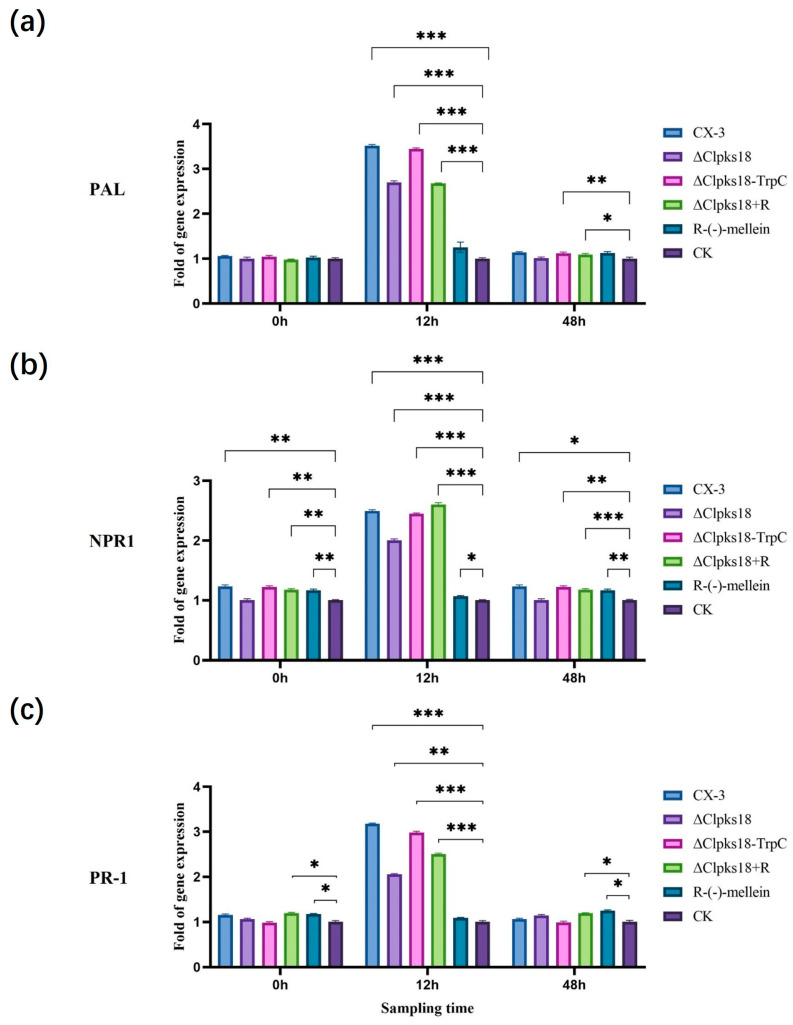
Effects of CLPKS18 on SA biosynthesis and signaling pathway. (**a**) Expression of gene encoding PAL in each treatment group. (**b**) Expression of gene encoding NPR1 in each treatment group. (**c**) Expression of gene encoding PR-1 in each treatment group. Figure note: +R indicates the addition of 0.8 ng/μL (*R*)-(-)-mellein in 100 μL. “*” indicates statistical significance (*p* < 0.05) using Tukey’s multiple comparison test. “**” indicates statistical significance (*p* < 0.01) using Tukey’s multiple comparison test. “***” indicates statistical significance (*p* < 0.001) using Tukey’s multiple comparison test. Bars represent the standard errors.

**Figure 6:**
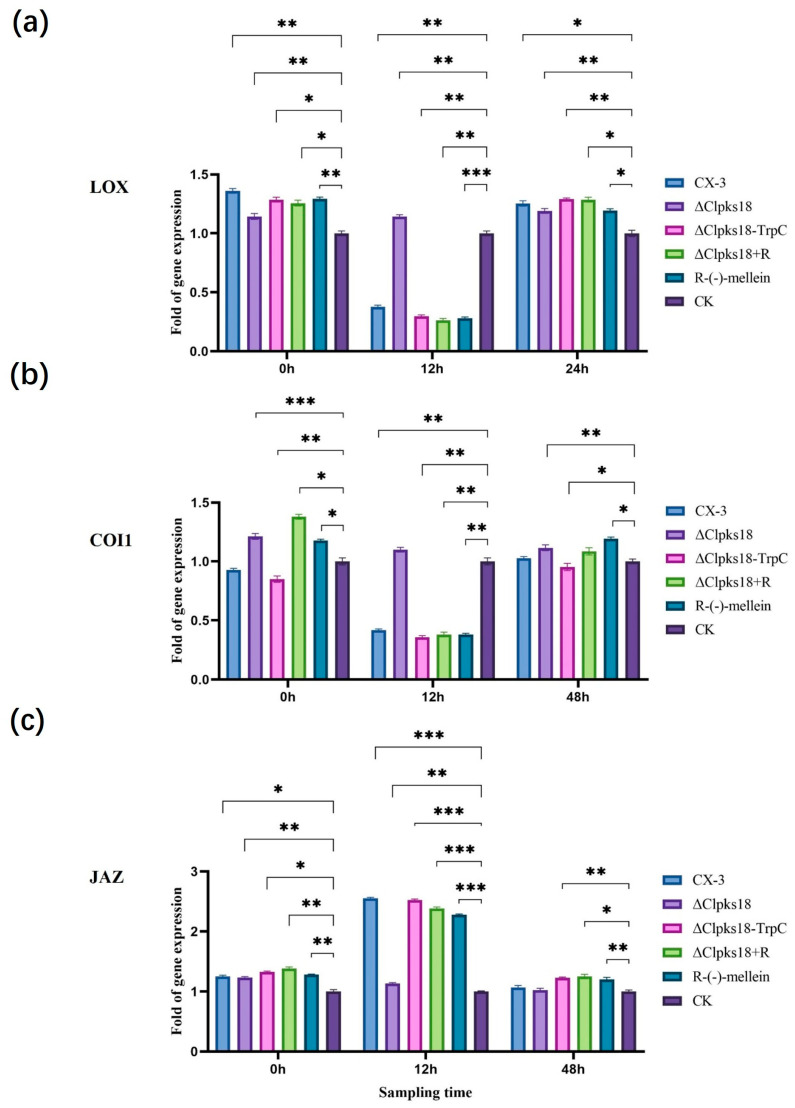
Effects of CLPKS18 on JA biosynthesis and signaling pathway. (**a**) Expression of gene encoding LOX in each treatment group. (**b**) Expression of gene encoding COI1 in each treatment group. (**c**) Expression of gene encoding JAZ in each treatment group. Note: +R indicates the addition of 0.8 ng/μL (*R*)-(-)-mellein in 100 mL. “*” indicates statistical significance (*p* < 0.05) using Tukey’s multiple comparison test. “**” indicates statistical significance (*p* < 0.01) using Tukey’s multiple comparison test. “***” indicates statistical significance (*p* < 0.001) using Tukey’s multiple comparison test. Bars represent the standard errors.

**Figure 7:**
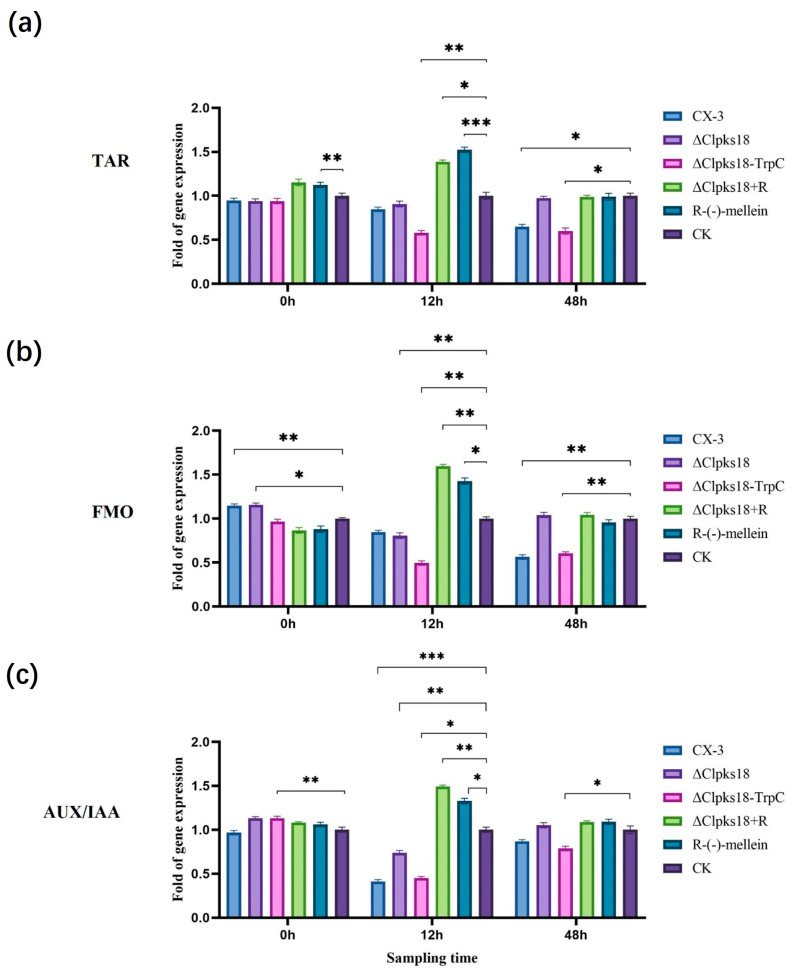
Effects of CLPKS18 on IAA biosynthesis and signaling pathway. (**a**) Expression of gene encoding TAR in each treatment group. (**b**) Expression of gene encoding FMO in each treatment group. (**c**) Expression of gene encoding AUX/IAA protein in each treatment group. Note: +R indicates the addition of 0.8 ng/μL (*R*)-(-)-mellein in 100 μL. “*” indicates statistical significance (*p* < 0.05) using Tukey’s multiple comparison test. “**” indicates statistical significance (*p* < 0.01) using Tukey’s multiple comparison test. “***” indicates statistical significance (*p* < 0.001) using Tukey’s multiple comparison test. Bars represent the standard errors.

The inoculated leaves were collected 0 hpi, 12 hpi and 48 hpi for RT-qPCR analysis for the quantification of the expression levels of different hormone-related genes in maize leaves pre- and post-inoculation. At 12 h post-inoculation, the expression of genes encoding PAL (LOC100384215) (a key enzyme for SA synthesis), NPR1 (a regulator on the SA signaling pathway) and PR-1 (LOC100286221) (a disease process-related protein downstream of the SA signaling pathway) was up-regulated more than 2-fold in CX-3-, Δ*Clpks18*- and C-Δ*Clpks18-TrpC*-inoculated maize leaves compared with the control (Figure 5a–c). LOX, a key enzyme for JA synthesis, and COI1 (LOC100280173), a positive regulator on the JA signaling pathway, were both down-regulated more than 2-fold, whereas JAZ (LOC100273108), a negative regulator, was up-regulated more than 2-fold (Figure 6a–c). In CX-3- and C-Δ*Clpks18-TrpC*-inoculated maize leaves, tryptophan aminotransferase (TAR; LOC103649700) and flavin-containing monooxygenase (FMO; LOC100037821), two key enzymes for IAA synthesis, were down-regulated, whereas the growth hormone-responsive protein AUX/IAA on the Aux signaling pathway was down-regulated more than 2-fold (Figure 7a–c). The expression levels of PAL, NPR1, PR-1, LOX, COI1 and JAZ were almost restored to the same levels as in the control group 48 hpi (Figure 5 and Figure 6). The gene expression of TAR, FMO and AUX/IAA (growth hormone-responsive proteins on the Aux signaling pathway) was still down-regulated in CX-3- and C-Δ*Clpks18-TrpC*-inoculated maize leaves (Figure 7). Overall, CLPKS18 was involved in regulating the pathogen’s interference with IAA synthesis and its signaling pathway in maize leaves; however, it did not significantly affect the synthesis of SA and its signaling process but stimulated the expression of genes related to JA signaling at some time points during pathogen infestation.

### 3.4. Comparison and Analysis of Effects of CLPKS18 and (R)-(-)-Mellein on SA, JA and IAA Production and Signaling Pathways in Maize

The leaves of the ZHENGDAN-958 maize seedlings in the five-leaf stage were inoculated with conidial suspensions (CX-3, ΔClpks18, C-Δ*Clpks18-TrpC* and 0.8 ng/μL (*R*)-(-)-mellein + Δ*Clpks18*) to determine the impact of (*R*)-(-)-mellein on the maize hormones related to the leaf defense response system. The inoculated leaves were collected 0 hpi, 12 hpi, 24 hpi and 48 hpi for UHPLC-MS analysis. It was observed that SA content and related gene expression showed no significant differences in the maize leaves treated with (*R*)-(-)-mellein relative to the control (CK) 12 hpi. However, there was a significant increase in SA content in maize leaves inoculated with CX-3, C-Δ*Clpks18-TrpC*, Δ*Clpks18* and Δ*Clpks18* + (*R*)-(-)-mellein (Δ*Clpks18* + M) (Figure 4a); further, some SA signaling pathway-related genes, i.e., *PAL*, *NPR1* and *PR-1*, were up-regulated two-fold compared with the CK group. At 48 hpi, the SA content in maize leaves inoculated with CX-3, C-Δ*Clpks18-TrpC*, Δ*Clpks18* and Δ*Clpks18* + (*R*)-(-)-mellein declined to that of CK (Figure 5). Thus, (*R*)-(-)-mellein was not a factor interfering with the synthesis of SA and its signaling transduction in maize. Regarding JA signal transduction, maize leaves inoculated with CX-3, C-Δ*Clpks18-TrpC* and Δ*Clpks18* + (*R*)-(-)-mellein, and those treated solely with (*R*)-(-)-mellein showed a significant decrease in JA content at 12 hpi (Figure 4b). Additionally, the key gene for LOX and the regulatory factor COI1 in the JA signaling pathway were down-regulated more than 2-fold, while the negative regulatory factor JAZ was up-regulated more than 2-fold. Furthermore, maize leaves inoculated with Δ*Clpks18* showed no significant difference in JA signaling pathway-related gene expression compared to the CK group. At 48 h after treatment with (*R*)-(-)-mellein alone, the JA content in the leaves was not significantly different from that of the CK group; this suggested that the inhibition of the JA signaling pathway by (*R*)-(-)-mellein was transient and mainly occurred in the early infection stage, between 12 and 48 h post-infection (Figure 6). Regarding IAA production, there was no significant difference in IAA content in Δ*Clpks18* compared to the control group (CK) at 12 hpi. However, the maize leaves treated with (*R*)-(-)-mellein and (*R*)-(-)-mellein + Δ*Clpks18* resulted in slightly higher IAA levels than CK over 12 h post-inoculation (Figure 4c). Similarly, the expression of the genes involved in IAA synthesis, TAR and FMO, as well as the AUX/IAA auxin response protein gene in the auxin signaling pathway, was up-regulated. On the contrary, the average IAA content in maize leaves inoculated with CX-3 and C-Δ*Clpks18-TrpC* decreased to approximately half of the CK level, and auxin synthesis-related genes such as TAR, FMO and AUX/IAA auxin response protein were all down-regulated more than 2-fold (Figure 7). Therefore, it was speculated that (*R*)-(-)-mellein and *Clpks18* negatively impact maize IAA production in different ways.

## 4. Discussion

The complex effects of CLPKS18 on *C. lunata* pathogenicity factors indicate diversity in the mechanism of disruption of maize leaf gene expression patterns. We performed dual validation with transcriptome analyses and RT-qPCR and found an interesting phenomenon: although CLPKS18 did not significantly affect SA synthesis and its signaling pathway in maize leaves, it significantly negatively regulated the expression of genes closely related to the IAA, SA and JA pathways (Figure 3, Figure 4 and Figure A1). This discovery indicates that CLPKS18 may affect maize growth, development and resistance to pathogen infection by inhibiting the signaling pathways of IAA and JA for maize growth and defense responses, respectively. Upon the induction of IAA, the early auxin-responsive gene families, including Aux/IAA, GH3 and SAUR, rapidly up-regulate their expression as a mechanism of response to the presence of IAA [16]. Similarly, it has been reported that tomato chlorosis virus (ToCV) infection or the stable transgenic overexpression of its p22 protein significantly decreases the expression of auxin signaling-responsive genes, suggesting that p22 can decrease host auxin signaling [17]. It has been reported that *Arabidopsis thaliana* over-accumulating the defense signal molecule salicylic acid (SA) frequently displays morphological phenotypes that are reminiscent of auxin-deficient or auxin-insensitive mutants, indicating that SA might interfere with auxin responses [18]. In maize, for example, when the maize growth hormone-regulated gene ZmAuxRP1 is highly expressed, root growth is accelerated, but resistance to stem and ear rot is declined, whereas ZmAuxRP1 positively regulates IAA synthesis but inhibits the synthesis of maize defense compound benzoxazinoids (BX) [19]. Thus, we hypothesized that virulence factors regulated by *Clpks18* may influence the balance between maize growth and immunity through some yet undefined mechanism.

Recent studies have revealed that PIF3 and PIF4 are not only transcription factors on the GA signaling pathway, but they are also directly involved in plant defense responses [20]. Specifically, PIF3 and PIF4 play key roles in signal switching between defense responses and normal growth in plants [21]. In this study, we are unable to determine with certainty whether *Clpks18* and its related secondary metabolites affect maize pathogenicity against *C. lunata* by affecting the GA pathway or whether they act directly by affecting PIF3 and PIF4. The role of GA in plant disease resistance or susceptibility mechanisms is complex and variable and depends on the pathogen and host plant species. For example, deletion mutations targeting the GA receptor gid1 in rice cause an increase in endogenous GA content, which in turn enhances resistance to *Magnaporthe oryzae* [22]. On the other hand, in maize, ZmGA2ox4, a member of the gibberellin 2 oxidase family, is responsible for regulating GA inactivation; its overexpression in maize resulted in a decrease in endogenous GA levels but an increase in resistance to *Fusarium verticilloides*, while exogenous GA treatment promoted maize growth but also increased susceptibility to *F. verticilloides* [23]. In rice, deletion mutations in *Eui*, an enzyme gene that regulates GA inactivation, lead to an increase in endogenous GA content, but this also makes rice more susceptible to rice leaf blight (*Xanthomonas oryzae pv. oryzae*) and rice blast (*M. oryzae*); however, the overexpression of *Eui* enhances resistance to these two diseases in rice. Further studies also showed that SA content decreased when endogenous GA synthesis was inhibited, while JA content was significantly reduced when gibberellins were synthesized in large quantities [24]. Based on the above studies, we can speculate that the virulence factor regulated by *Clpks18* in *C. lunata* affects the maize GA signaling pathway; it may likewise interfere with the expression of genes related to the maize JA/SA signaling pathways.

We determined the changes in the contents of SA, JA and IAA in maize leaves at different infestation times and observed that CLPKS18 mainly affected the production of JA and IAA in host leaves. In the JA signaling pathway, the expression of COI1 was repressed, while the expression of JAZ was up-regulated. JAZ, as a transcriptional repressor in the JA signaling pathway, was able to efficiently inhibit the transcription process of the genes related to the JA signaling pathway [25,26,27]. This study revealed that CLPKS18 inhibits gene expression in the JA signaling pathway by activating JAZ proteins in leaves.

(*R*)-(-)-mellein significantly inhibited JA synthesis and its signaling pathway 12 to 48 h after maize was infected by *C. lunata*. However, on the 48th hpi, the JA levels were gradually restored to untreated (CK) levels, which revealed a temporal effect of (*R*)-(-)-mellein during pathogen–host interactions. We speculated that (*R*)-(-)-mellein may promote pathogen colonization during the early stages of *C. lunata* infection by diminishing the interference of host defense responses to pathogen colonization on leaves. (*R*)-(-)-mellein stimulated IAA accumulation in maize leaf to some extent compared with CK post-inoculation, which indicates that the overproduction of IAA in infected leaves could be viewed as a susceptible response. Early work has demonstrated that the *Pseudomonas syringae* strain DC3000 produces auxin as a virulence factor to suppress SA-mediated defenses in *Arabidopsis thaliana* [28,29].

(*R*)-(-)-Mellein has also been isolated from *Stagonospora apocynin*, the causal agent of leaf spot disease in cannabis (*Apocynum cannabinum* L.), where it functions as a phytotoxic metabolite inducing leaf necrosis in *Setaria faberii Herrm* and eight other weed species [30]. This compound is recognized as a pathogenic factor in *Botryosphaeria dothidea*, which secretes it into host tissues during fruit and branch infestation. A prior study proposed a correlation between the expansion of disease lesions and the production kinetics of (*R*)-(-)-mellein in apple tissues [8]. Additionally, (*R*)-(-)-mellein was detected in grapevines infected by *Diplodia seriata*, with its concentration showing a positive correlation to the pathogen density within the host plants [31]. This study for the first time reveals the non-cytotoxic mechanism of (*R*)-(-)-mellein in maize: this compound specifically inhibits the jasmonic acid (JA) signaling pathway to weaken plant defense responses, as evidenced by down-regulated expression of LOX and COI1 genes and up-regulated expression of JAZ genes. Concurrently, it transiently promotes indole-3-acetic acid (IAA) accumulation to enhance plant growth signaling (Figure 6 and Figure 7). This mechanism differs significantly from the role of (*R*)-(-)-mellein as a simple toxin in *Nectria fuckeliana* [6], indicating that this compound may employ diversified pathogenic strategies in different host–pathogen interactions.

JA is the core hormone for plant antifungal defense. The continuous inhibition of its key synthesis enzyme LOX and positive signal regulatory factor COI1 (Figure 6a,b) directly weakens the immune response of corn to *C. lunata*, which is consistent with the mechanism by which pathogenic bacteria in *Arabidopsis thaliana* enhance pathogenicity by inhibiting the JA pathway [32]. *Clpks18* inhibits host growth by down-regulating the IAA synthase TAR/FMO and the response protein AUX/IAA, while (*R*)-(-)-mellein can temporarily promote IAA accumulation (Figure 7a,c). This contradictory phenomenon may stem from the manipulation of the “growth-defense” resource allocation of plants by pathogenic bacteria, inducing susceptible phenotypes through local increase in IAA, while systematically inhibiting growth to deplete host energy [19]. The up-regulation of SA content and related genes (PAL, NPR1) in the early stage (12 hpi) treated with CX-3 and (*R*)-(-) -mellein (Figure 5a,b) might be a “pseudo-defense” response triggered by pathogenic bacteria, which antagonizes the subsequent JA-dependent defense by consuming SA synthesis precursors, similar to the strategy of viruses in tomatoes promoting infection by manipulating the SA-IAA balance [17].

## 5. Conclusions

This study demonstrates that *C. lunata* employs the *Clpks18* gene and its biosynthesized secondary metabolite (*R*)-(-)-mellein to execute a sophisticated hormonal interference strategy during maize-*C. lunata* interaction. The pathogen effectively dismantles host defense systems through temporally regulated modulation of core phytohormone pathways: significantly suppressing JA signaling (manifested as down-regulation of LOX/COI1 and 50% reduction in JA content), implementing paradoxical yet synergistic regulation of IAA metabolism, and selectively inhibiting GA-responsive factors PIF3/PIF4. Crucially, (*R*)-(-)-mellein exhibits dual functionality—simultaneously suppressing JA signaling and amplifying IAA modulation—revealing a novel mechanism of pathogen-mediated plant physiological manipulation. These coordinated disruptions of JA/IAA/GA pathways facilitate fungal invasion by compromising maize defense. Future work should enhance JA pathway signaling expression in maize germplasm against pathogen (*R*)-(-)-mellein interference. These findings not only deepen our understanding of *C. lunata* pathogenesis mechanism but also provide actionable targets for disease control through PKS inhibitor development and JA-enhanced germplasm screening. Future research can mainly focus on these areas: A. Gene Function Verification: Create maize COI1 and AUX/IAA mutants and combine them with *C. lunata* inoculation experiments to clarify the roles of key nodes in the hormone pathway. This will help us understand how these genes contribute to the plant’s response to the pathogen. B. Protein Interaction Analysis: Screen host proteins that bind to (*R*)-(-)-mellein to find its molecular targets, such as the JAZ protein. Uncovering these interactions is key to understanding the molecular events during the plant–pathogen encounter. C. Hormone Transport Network: Track the transport routes of SA, JA, and IAA in maize to see if CLPKS18 disrupts the transport to reshape systemic defense. This research will shed light on the plant’s long-distance defense coordination.

## Data Availability

The data (The transcriptome and metabolome data) presented in this study are currently held by the authors and have not yet been uploaded to public databases. They will be made available upon reasonable request to the corresponding author.

## Appendix A

**Table A1:** Primers for assays of gene expression related to JA, SA and IAA synthesis and signal transduction.

Primer Name	Sequence (5′ to 3′)
LOC100274569F	TGAGTACCGCAAGGATGGTG
LOC100274569R	GCGCGACGATACACTAGACA
LOC103641361F	CCCGAACATTCCCGTTTGGT
LOC103641361R	AAGGACTCTGTGCCGTTTGG
LOC100384229F	TGACGAAAATCCAGCCAGCA
LOC100384229R	AGCAGGAAATGGTGTTAGCGA
LOC100193303F	TCCAGATCCTCTCCGCCAAC
LOC100193303R	TGCAAACAGGCACAAATGCG
LOC103653849F	TACTTCAACCACCCGCTGT
LOC103653849R	CACATGCTGCTACCACTACCA
LOC103649700F	GTCACGGATTTCCTCCGGTC
LOC103649700R	TGATGGCTGTGTACTGCGG
LOC100037821F	CCTTGGTTATCCGTAGCCCG
LOC100037821R	CTTGGCCTTGAGAAGAAGCG
lox1f	GACCCTCGCCTCAAGAACC
lox1r	GAACGGGGAAACGCAAACAA
LOC100273108F	CCCAGTTCTCCTCCTTCGAC
LOC100273108R	AGCTCTGGACCCTGAACATC
LOC103638892F	CCGTTCTTCAAGCCCGAGTC
LOC103638892R	GTGGTGGTTGGTGTTGCTCG
LOC100280173F	CCGGCTTGTTCTGCTCGATA
LOC100280173R	CATCATCGGTTTCCCCAACG
LOC100384215F	GCGAGAAGCTCAAGTCCCC
LOC100384215R	TGTCATCTCCTCCTCTTGCG
LOC100281196F	TCGCCTTTGTTGGCAGATGA
LOC100281196R	CTGGCGTCTCATTAAGGTCCA
LOC100286221F	AGATCATGTGGCGGAGCAC
LOC100286221R	CAGCAACGATTTGGGACAGC
ZmUBI-F	GGAAAAACCATAACCCTGGA
ZmUBI-R	ATATGGAGAGAGGGCACCAG
